# Aging and Caring: Exploring Older Adults’ Motivation for Informal Caregiving to Other Aging Individuals in Nigeria

**DOI:** 10.1093/geroni/igad140

**Published:** 2024-01-02

**Authors:** Juliet Chigozie Donatus Ezulike, Shiyu Lu, Marcus Yu Lung Chiu

**Affiliations:** Department of Social and Behavioural Sciences, City University of Hong Kong, Kowloon, Hong Kong, People’s Republic of China; Department of Social Work, University of Nigeria Nsukka, Nsukka, Enugu, Nigeria; Department of Social and Behavioural Sciences, City University of Hong Kong, Hong Kong, People’s Republic of China; School of Health & Wellbeing, University of Bolton, Bolton, Manchester, England; Felizberta Lo Padilla Tong School of Social Sciences, Caritas Institute of Higher Education, Tseung Kwan O, Hong Kong, People’s Republic of China

**Keywords:** Caregivers, Culture, Motives, Nigeria, Oldest-old

## Abstract

**Background and Objectives:**

Because of the global population aging, more informal carers become older adults. In Nigeria, the African country with the largest population of adults aged 60 years and older, self-construal rooted in the African collectivist philosophy generally shapes informal caregiving for older adults. However, there is a general paucity of studies on older adults’ informal caregiving roles, particularly about their motivations for caregiving. This study explored older adults’ motives for informal caregiving to their care recipients in urban Southeast Nigeria.

**Research Design and Methods:**

This study adopted a hermeneutic phenomenological research design. In-depth interviews were conducted with 30 purposively selected older adults aged 54–88 who were the primary carers of other older adults in the family and community. The collected data were analyzed using van Manen’s thematic analysis method, using QSR NVivo 12 software.

**Results:**

A total of 4 main themes emerged from the participants’ responses: reciprocity of kindness, altruism, a sense of moral responsibility, and eagerness for peaceful longevity. The findings generally showed that religion and culture were the latent factors ingrained in these motivations for informal caregiving.

**Discussion and Implications:**

Although the African philosophy emphasizes altruism, reciprocity seems more prominent in specific traditional African communities, as observed in Southeast Nigeria. It serves as a means to prioritize family members’ needs. The findings indicate the need for the government to establish sustainable programs and policies that support older people in their caregiving role. Doing so will enable carers to derive psychosocial gains from informal caregiving and sustain the caregiving culture of Nigeria.

**Translational Significance:** This study investigated older adults’ motivations for caregiving to other older adults such as family members and friends in Southeast Nigeria. The motivations were attributed to reciprocity, altruism, a sense of responsibility, and an eagerness for peaceful longevity. We recommend future studies investigating this topic among older caregivers in other regions of Nigeria. Studies should also explore younger caregivers’ caregiving motives, as the dominant factors among youths may differ from those of older carers. Findings from such studies can shape Nigeria’s welfare policies and enable this cohort of older carers to prepare better for their later life care reception.

## Background and Objectives

Globally, informal caregivers are fundamental in caring for sick and disabled individuals. However, caregiving, especially for older adults and adults with chronic health conditions, has become a significant global concern (1,2), with its value often challenging to measure due to its unpaid nature (3). In 2005, global care costs were estimated at US$315 billion, with two-thirds falling on health care budgets and the third being the financial and social burden on unpaid informal caregivers (4). In higher-income countries, the demand for caregiving is driven by increased longevity and the rising cost of formal care, among other factors (5,6). Consequently, older adults constitute a significant proportion of informal carers in aging societies (7,8).

Conversely, in low-and-middle-income countries, especially sub-Saharan African (SSA) countries like Nigeria which has a high youth population and relatively low life expectancy (52.6) (9,10), informal carers are mainly young and middle-aged. Evidence suggests that early marriage, especially among women, and high fertility are major drivers of the predominance of youthful caregivers in SSA (11), implying that SSA in later life are more likely to have mid-life-aged children who are closer in age to them. Within this region, the absence of formal support and cultural prescriptions about aging family members influence informal caregiving demand (12–14).

Existing studies have established that different motives may drive informal caregiving. Motivations for caregiving generally refer to the reasons that underlie, promote, and sustain informal care and support provision. These motives include reciprocity (15–18), spirituality and religion (19–25), empathy (18), financial reasons (25), affection (26), and cultural reasons (eg, filial piety among the Chinese, Thai, Korean, and Taiwanese caregivers (22,27)), birth position in the family among Japanese, Turkish and Moroccan caregivers (22,28), and the sense of obligation and responsibility (15,18,29).

In Nigeria, self-construal rooted in the African collectivist philosophy generally shapes informal caregiving. Among the provisions of the African philosophy is that caregiving is older people’s cultural right as they are considered the representatives of ancestors, creators and custodians of cultural traditions, and mediators between the physical world and the one beyond (30). However, recent studies report that informal caregiving in Nigeria is increasingly challenging due to economic decline, lack of government support, and adverse caregiving impacts on carers (2,12,31–34). Despite these challenges, many individuals continue taking on the caring role, leaving much yet to be known about their underlying motivations.

Understanding the cultural factors that shape informal caregiving is crucial, and empirical studies play a vital role in unraveling the nature and extent of such influence. The existing global caregiving literature identified various caregiving motives. However, they were predominantly studied in the context of exchanges between aging parents and their adult children, overlooking how the form of caregiver-care recipient relationship may drive different caregiver groups’ motivations. In the Nigerian caregiving literature, 1 study highlighted reciprocity’s role in intergenerational exchanges between older adults and their adult children in northern Nigeria (16), while most other studies focused on caregiving’s adverse impacts, emphasizing that it leaves negative outcomes on caregivers’ health, finances, and overall wellbeing (31,33,35–38). Moreover, by intentional or accidental design, existing studies had more samples of young and middle-aged caregivers below 50 years, thus limiting insights from older informal caregivers.

Reasons for the lack of research focus on older carers may include difficulty in accessing them especially if they reside in remote areas, and researchers’ non-realization of the rate at which young and middle-aged caregivers are maturing into the status of older adults in Nigeria. Thus, to the best of our knowledge, this study is the first to focus on this specific caregiver group and is part of a more extensive study on older Nigerian adults’ informal caregiving experiences. This study empirically explores older Nigerian adults’ motivations for informal caregiving to other aging individuals in their families and communities.

## Theoretical Framework

This study is based on the theory of interdependent selfways which explains that individuals in collectivist cultures prioritize group harmony, interdependence, and social connections (39). It highlights the significance of culture in shaping individual behavior, cognition, self-perception, and interactions with others. This theory differs from other theories explaining cultural factors that shape motivations, given its applicability within various collectivist cultural contexts and its focus on psychological mechanisms underlying cultural values and interdependence. In the context of this study on older adults’ motives for informal caregiving, the theory offers insight into the cultural foundations of caregiving in Nigeria, reflecting how engagement in some culturally patterned practices shapes psychological habits and tendencies.

In traditional African ethics, harmony is a revered virtue. Based on this virtue, important exalted values include sympathy, beneficence, altruism, generosity, tolerance, and compassion (40). These virtues are also associated with the Ubuntu philosophy, which emphasizes the interconnectedness of humanity, asserting that individuals are shaped by their relationships and responsibilities to the community (41). According to this perspective, an individual has some obligation to care for anyone in need, including strangers. However, having personal or blood ties with the individual plays a major role in considering which of those in need takes priority and how much aid to avail them (29,42).

Additionally, respect for elders and ancestors is deeply ingrained in African ethics, where the wisdom and guidance of older generations are greatly esteemed (30). An individual’s self-construal is thus shaped by their intergenerational solid connections and the obligations they feel to honor and preserve ancestral legacies. In this study, we leverage the theory of interdependent selfways to explore how cultural norms and relationships shape older Nigerian adults’ informal caregiving motivations, thus contributing valuable insights to caregiving research in Nigeria.

## Research Design and Methods

### Research Design

We adopted van Manen (43) hermeneutic phenomenological design in this study. This design facilitates understanding the phenomenon under study by reflecting on the participants’ experiences of informal caregiving based on their prevailing cultural and social contexts.

### Setting and Participants’ Recruitment

This study was conducted in Owerri, the capital city of Imo State, Southeast Nigeria. In 2006, the census record of the city’s population was 403 425 (197 944 men and 205 481 women). Nevertheless, with an average annual population growth rate of 4.05%, the city’s population in 2020 was estimated to be 872 604 people (44). Like other parts of Southeast Nigeria, the indigenes of Owerri are of the Igbo ethnic group. The Igbo people have a strong tradition of caregiving, rooted in the belief that older adults are respected members of society and hold important societal roles. Imo state has a high percentage of the literate population (45,46), with locals known for their dedicated work ethic across various sectors such as agriculture (in rural areas) or services/industry/commerce (in urban areas) (47).

Due to initial difficulties in recruiting participants, snowball sampling was used to recruit a convenient sample of 30 informal caregivers aged 54–88 years. The initially identified participants recruited from the first author’s network were requested to recommend other participants. For every recommended participant, a phone call was made to introduce the research team, informing participants about how their phone contact was obtained, and acquaint them with the purpose and requirements of the study. A total of 35 potential participants were contacted. However, 5 individuals, including 2 men and 3 women, declined participation in the study. Age 54 was used as a cut-off old-age marker in this study based on the commonly used age markers (50–55 years) in gerontological studies conducted within SSA (48–51).

### Data Collection and Analysis

Between July and November, 2021, we conducted 26 telephone and 4 face-to-face interviews, lasting for 50–75 min. See the sample interview guide in Supplementary Material. The data collection tools were an interview schedule for keeping track of questions and an audio recorder to facilitate data transcription. The interview data were first transcribed verbatim and pseudonymized to aid data analysis. Twenty-nine interviews were conducted in English and 1 in a mixture of English and Igbo. With all transcriptions performed by the first author, the interview in Igbo was translated to English using the parallel transcription framework (52), while an independent researcher proficient in English and Igbo validated the translation thereafter.

**Figure 1.**
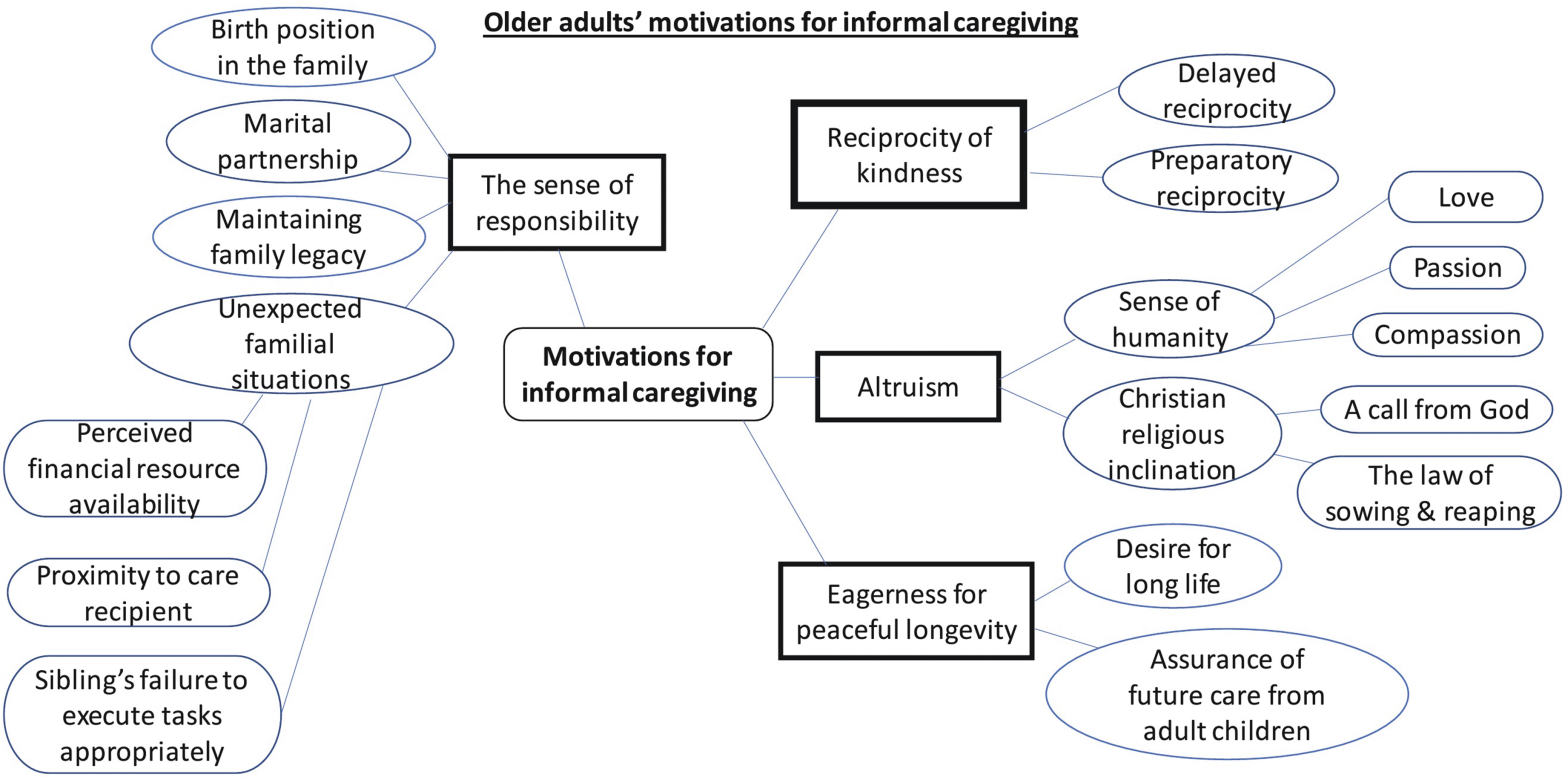
Thematic map.

During the data analysis, we utilized 3 techniques for thematic analysis of phenomenological studies, as described by van Manen (1997). First is the holistic approach, which involves reading each transcript to understand its meaning. Second is the selective approach, identifying meaningful parts of the transcript texts. The last is the line-by-line approach that enables a systematic questioning of the data concerning the respondents’ caregiving experiences. Listening to the participants’ voices during the data analysis allowed us to examine the patterns, connections, and common themes that describe their motivations for caregiving. We drew a thematic map (see Figure 1) to help us understand the relationships among codes and the connections among themes (53). Meetings were scheduled to enable us to discuss, review, and refine the themes. Additionally, member checking was employed to verify the credibility of our findings (54). Ethical approval for this study was received from the City University of Hong Kong’s College of Liberal Arts and Social Sciences human subject ethics sub-committee.

## Results

A summary of the study participants’ demographic information is shown in Table 1 while that of their care recipients is shown in Table 2. Different reasons and situations made the study participants assume the caregiving role. Four broad themes emerged from the responses of informal caregivers in this study. These themes are (a) reciprocity of kindness, (b) altruism, (c) the sense of moral responsibility, and (d) eagerness for peaceful longevity.

**Table 1.** Demographic Information of Informal Caregivers (*N* = 30)

Variable	*n* (%)
Age	
54–59	21 (70.0%)
60–69	7 (23.3%)
70–79	1 (3.3%)
80–89	1 (3.3%)
Gender	
Men	14 (46.7%)
Women	16 (53.3%)
Education	
Primary/secondary education	1 (3.3%)
Undergraduate	15 (50.0%)
Postgraduate	14 (46.7%)
Residence arrangement	
Co-resident caregiver	14 (46.7%)
Non-co-resident caregiver	16 (53.3%)
Number of care recipients	
One care recipient	27 (90.0%)
More than one care recipient	3 (10.0%)
Health status	
Fair	11 (36.6%)
Good	19 (63.3%)
Marital status	
Married	23 (76.7%)
Widowed	6 (20.0%)
Separated	1 (3.3%)
Relationship with the care recipient	
Spouse	4 (13.3%)
Adult child	17 (56.7%)
In-law	8 (26.7%)
Friend	1 (3.3%)
Employment status	
Employed	25 (83.3%)
Retired	5 (16.7%)
Duration of caregiving	
1–11 mo	2 (6.6%)
1–5 y	12 (40.0%)
6–10 y	9 (30.0%)
11–15 y	7 (23.3%)

**Table 2.** Demographic Information of Informal Caregivers’ Care Recipients (*N* = 33)

Variable	*n* (%)
Age	
54–59	1 (3.0%)
60–69	4 (12.1%)
70–79	3 (9.1%)
80–89	16 (48.5%)
90–99	9 (27.2%)
Gender	
Men	14 (42.4%)
Women	19 (57.5%)
Health status	
Fair	6 (18.1%)
Poor	10 (30.3%)
Frail	17 (51.5%)

### Reciprocity of Kindness

This theme denotes the need to return various acts of kindness through informal caregiving. Reciprocity in the context of the caregivers’ responses may be perceived as the felt obligation to return a benefit in cash and in-kind. It manifests in the forms of delayed and preparatory reciprocity.

#### Delayed reciprocity

Delayed reciprocity is when individuals try to return to a benefactor the acts of kindness they received in the past. For many participants in this study, past indebtedness is cited as a motivation for caregiving and was commonplace amongst all the different forms of carers. Elizabeth, a 56-year-old spousal caregiver, shared how her husband’s selflessness and unwavering dedication inspired her to take on the caregiving role. Initially hesitant to pursue further schooling due to being an orphan and feeling the responsibility toward her younger siblings, her husband encouraged her to become a teacher, providing financial assistance for training forms and continued support throughout their journey. Even as they became parents, he actively participated in childcare duties and facilitated school transportation. She commented:

I benefited a lot. He [care recipient] married me the year I finished my school cert … I didn’t want to go to school. I wanted to start doing business so that I can train my siblings. But my husband refused. He said he has been desiring his wife to be a teacher. Then, with that, we bought the form for teacher training, and they gave me admission. He sacrificed a lot …, taking care of our 3-month-old baby while I attended school. When I finished that teacher training, … he bought me form for sandwich program … He was paying my school fees, not minding that I was working. He was a father, a brother, a friend, and everything for me.

Some carers also described deep-rooted perceptions that the Igbo culture involves reciprocity. Such is made manifest through cultural role expectations from caregivers. The participants revealed that due to their culture, they grew up knowing they had to assume the caregiving role as reflected in the following statements:

I believe it is my duty to do so [caregiving]. Probably because of how one is socialized… I have always known that reciprocity is part of Igbo culture. And this is something I have known all my life. It did not start today. For instance, you’ll see somebody giving out his daughter in marriage, and people bringing him drinks because he gives to others in their time. So, reciprocity guides a lot of things we do in Igbo land. (Charlotte, 54, Daughter).

You see, it [caregiving] is an innate behaviour. You’re not really paying back. You’re growing up to know that already. In our Igbo parlance, we say “When the mother is through training the child, the child, in turn, trains the mother.” (Raynox, 54, Son).

#### Preparatory reciprocity

Although delayed reciprocity was the main form of reciprocity as a motive for caregiving, we also found preparatory reciprocity as some participants’ motivation for caregiving. In this study, some participants assumed the caregiving duty to show their adult children what to learn from them on how to provide quality care for them later in life. The caregivers tried to serve as a good example to model the right attitude to informal caregiving for their adult children to emulate later. Clara, a 56-year-old adult-child explained:

You know, when you care for your parents and the younger ones are watching you, they grow up to begin to care for you too. For instance, this morning my son came and said, “We need to change something in your house.” I didn’t tell him or any of my children I needed that change. But because they look at me taking care of my mother and father, they are watching you and imitating … When you don’t care for your own parents, you don’t expect the younger ones to care for you.

### Altruism

This theme describes the decision to provide informal care resulting from empathy and concern for the welfare and happiness of others to give them a quality life. Altruistic motivations for care provision were made manifest through caregivers’ emotions of humanity and Christian religious inclination.

#### The sense of humanity

The findings showed that assuming the caregiving role is driven by participants’ intense emotions that depict humankind, such as love, compassion, and passion for older persons. Participants mentioned that a common motive for caregiving is affection for their care recipients. Such motivation was common among adult-child caregivers primarily when they reminisced about their childhood/youthful years. Love was signaled through financial/material provisions to care recipients:

So, it’s like a case of love begetting love, and you’re doing it [caregiving] whether you’re asked to do it or not. You’re doing it and then you don’t even believe that somebody else can do it better than you. So, it’s not like they [care recipients] are asking. But you’re giving and you’re happy giving the care. (Joycelyn, 60, Daughter).

Also, it was evident in their responses that caregiving was driven by compassion following care recipients’ sufferings. Compassion was evident when care recipients experienced debilitating health conditions that robbed them of the capacity to assist themselves. Johnson, a 62-year-old spousal carer said:

I know that her [care recipient] condition requires pity … Somebody you have been living with, who was sound in health is now suddenly being carried about. It is something that is difficult for one to comprehend. So, I look at it from the angle of pitying her, and that made me not to even scold her when she makes a mess … I saw what she was passing through and it wasn’t a joke.

Similarly, carers suggested it is natural for them to help older people in need regardless of whether they are related. Annis, a 62-year-old Daughter-in-law noted, “There is this kind of passion I had. I had passion for the elderly from my childhood because, I lived with my grandaunt. So, you know, it became part of me to take care of the elderly.” This intense feeling about the need to help older people was more common among female carers than male carers. It was usually the case if they grew up or spent a significant part of their childhood and teenage years with older adults.

#### Christian religious inclination

The study participants identified as Christians. Hence, many were driven by the tenets of their religion, citing that the Bible admonishes adherents to care for people in need. One of the ways in which the religious rationales for caregiving manifested was by perceiving caregiving as God’s call. Carers who felt that God had called them to service believed that such a calling gave them the right attitude to caregiving, irrespective of other commitments they might have. In the words of Kendra, a 54-year-old adult-child:

I take it as a call from God. I just decide to see it that way. I see it as an understanding, more like. You know everybody is very busy, everybody has their issues and it’s not everybody that has the right attitude to challenges in life. So, it is more like an attitudinal thing. Let me say maybe I have a better understanding towards accommodating. That’s the way God made me, and I have to fulfil what God wants me to do. So, I just leave it at that, I don’t want to put anything special to it.

Again, some participants attributed their caregiving motives to the Christian parable of sowing and reaping, which depicts the law of karma in other contexts. They noted that their assistance to their care recipients guaranteed their receipt of caregiving from various persons in late life. This idea was observed among participants who helped an in-law or a friend:

Being a spiritual person, life is a seed. If you sow, you will reap. As I provide care for this person at this level, If I find myself in this state later in my life, which obviously will come, I will not lack care. Because I have sown the seed now, definitely, I will reap it. (Jack, 54, Friend).

### The Sense of Moral Responsibility

This theme implies caregiving being motivated by the felt sense of obligation toward older adults needing care. Some carers assumed the caregiver role due to their sense of obligation toward their care recipients. This sense of obligation was made manifest in different contexts and situations.

#### Birth position in the family

One’s birth position in the family was a unique factor that conferred caregiving obligation among some study participants. Depending on the prevailing practice in their local village communities, being the first son, first daughter, last son, or the last daughter meant that they were primarily responsible for the informal care of their parents. Charity, a 57-year-old daughter said:

According to my tradition, I am the person to look after my parents. They said as the last born, I am supposed to be the one close to my parents and take care of them in their old age. As we were growing up, my siblings were leaving the house one after the other. And at that point, I was the last of the girls. So, the whole thing [caregiving responsibility] turned to me and I had to stay with them.

#### Marital partnership

Among spousal carers, 1 of the motivations for informal care was the sense of obligation toward their spouses due to their marital vows and existing collaboration over the past years. Such a sense of duty was regardless of whether caregivers have had a cordial spousal relationship with their care recipients. Nora, a 56-year-old spousal carer said, “He is my husband and I promised him the day we met at the alter that I will take care of him in health and in sickness.” Another spouse, Johnson, said:

In my own case I was looking at it as my responsibility, whether we were in good terms or not. Somebody is your partner, you are living with the person, and she has children; your children and her children who live with you. When they look up to you, how do you abandon their matter? We’ve been doing the same thing together for years … She was working and was bringing in finance. She was contributing to the training of the children…We ran the home together.

#### Maintaining family legacy

A recurring motivation for caregiving in many Southeast Nigerian communities is the avoidance of social repercussions, including stigmatization, ostracism, and social isolation typically targeted at families who fail to cater to their older adults. Patience, a 65-year-old, extended family member explained that she assumed the informal caregiving duty to her husband’s aunt after her husband’s demise. The role assumption was to continue her late spouse’s caregiving legacy and present a good image of her immediate and extended family before the local community:

She [care recipient] hasn’t done anything for me. Only because I am married to that family. You have to protect the name of your husband. If your children go out and do something bad, it’s your surname they [outsiders] will be calling, you see. So I am not doing it [care provision] because of what I gain from them, I don’t gain anything.

#### Unexpected familial situations

Certain occurrences within the family system could inform the sense of moral responsibility concerning the need to provide informal care. Such situations may have cultural, social, or economic significance. One such situation is related to financial resource availability. Especially among participants assisting their parents or parents-in-law, this study revealed that the caregiving role was inevitable because they possessed higher financial resources than other family members:

I’m not the Ada (first daughter). So, it depends on the heart and resources… It is a decision you actually take … I really feel like taking care of her [care recipient]. I know what she needs, you know. So, that is it … I’m more responsive and I got resources [employment] before every other person. So, that responsibility now automatically fell on me. (Catherine, 55, Daughter).

Also, it was evident from some responses that the caring role was inevitable for individuals in proximity to their care recipients. When older persons have their younger kin residing in faraway locations, those closest to their location most often become their primary informal carers. Charlotte, a 54-year-old daughter explained:

I am their [care recipients’] only daughter, and I have two brothers who are not like family-strong, so to say. And incidentally, none of them lives in Nigeria. So, it wasn’t like there was an option or something. There was no option.

Similarly, some participants’ responses revealed that the caregiving role was motivated by some family members’ failure to discharge the caregiving duty satisfactorily. Without proper caregiving, their care recipients will have numerous unmet needs, possibly resulting in the entire family being subjected to public ridicule in the community. Consequently, the defaulting individual rescinds from the primary caregiver role for other persons to assume as a matter of necessity. Frank, a 56-year-old son shared his experience of taking on the role of caregiver after his elder sister as the primary caregiver and her adult children were unable to perform their responsibilities adequately:

They’ll [elder sister and her children] help themselves with some of the money we contribute, and at the end of the day, the money won’t be enough to take care of my father. The last straw that broke the camel’s back was when we employed an informal carer from XX state to stay with my father. If we [younger siblings] send money to these people to pay the carer, they won’t pay him. So, the person had to leave. At that point, I took up the caregiving responsibility.

### Eagerness for Peaceful Longevity

The eagerness for peaceful longevity is a unique finding in this study. As older adults are held in high esteem in the caregivers’ culture, the attainment of old age is usually celebrated, with the family and community availing them of care and support. This theme explains caregivers’ assistance to older adults due to certain beliefs engrained in the Igbo culture, suggesting a challenge-free life in old age.

#### Desire for a long life

Some caregivers were motivated to assist their care recipients because they hoped to live a long life. In traditional Igbo society, older adults are accorded great respect and specific responsibilities based on the idea that they possess wisdom. In many cases, it is believed that the longevity of the care recipient will be replicated in the caregiver of an older adult. Raynox, a 54-year-old son commented, “I want her [mother] to live as long as possible. If she has a long life, it will make me also have a long life.”

#### Assurance of future care from children

Many caregivers continued in the caregiving role due to the assurances their adult children provided about such care in the future, construing it as a cultural right and God rewarding these caregivers, now and then, with a well-catered later life. Jane, a 63-year-old daughter-in-law said, “Somethings could happen, and my son will tell me ‘Ah! Mummy, this thing should not bother you. As you are taking care of her [care recipient], my wife will equally take care of you.’ You understand.”

## Discussion and Implications

### Discussion

The main aim of this phenomenological study was to investigate older adults’ motivation for informal caregiving, drawing on the theory of interdependent selfways. According to our study’s findings, reciprocity emerged as a major motivation for caregiving. Specifically, delayed reciprocity was shown to be the primary caregiving motive. This form of reciprocity involved participants aiming to repay their care recipients for past benefits they had received from them. Similar findings have also been reported in other studies (15–18). Additionally, we identified preparatory reciprocity as a caregiving motivation wherein carers relied on this form of reciprocity by demonstrating appropriate caregiving behavior to their adult children, hoping they would observe and emulate it later. This outcome has also been observed in previous studies such Ugargol and Bailey (17) work conducted in India.

Although research suggests that reciprocal motivations exist among informal carers across different cultural contexts globally, this study highlighted specific cultural connotations associated with reciprocity in Southeast Nigeria. The participants in this study expressed that reciprocity played a significant role in their culture, as they were socialized to prioritize their care recipients’ needs through this practice. This finding supports Bell and Metz (42) assertion that individuals with Afrocentric self-construal prefer those with whom they share personal or blood ties when determining who should receive aid and how much assistance should be provided. This cultural connotation also manifested in the form of preparatory reciprocity where informal caregivers shaped their caregiving preference through demonstration, as the expectation of informal care from one’s children is inherent in the African philosophy.

However, preparatory reciprocity, as expressed in this study, implies the participants’ expectation of receiving similar quality of caregiving from their adult children. Yet, the increasing unemployment and underemployment leading to the experience of material/financial limitations among young Nigerian adults (9) makes the realization of this expectation uncertain. Consequently, there is a pressing need for social policies supporting informal caregivers across Nigeria’s general population.

Aside from reciprocity, caregiving motivations included altruism. Altruistic caregiving was driven by the sense of humanity, which involved humanistic emotions of affection, passion, and compassion. Such humanistic emotions have been documented in a recent study conducted in Ghana (18), with affection being associated with financial and material provisions as demonstrated in Coe (26) study of Ghana. These humanistic emotions align with the Ubuntu philosophy which shapes the interdependent self-way of people in diverse African communities (41,55). The uniqueness of the interdependent self-ways that drive behaviors in African communities lies in its ability to manifest these harmonious values without restrictions regarding location or individuals involved, thus setting apart African philosophy within collectivist cultures.

Altruistic caregiving motivations were also present among the caregivers following their Christian religious inclination, as observed in construing caregiving as God’s call and the act of sowing and reaping. Such religious notions have been noted in previous studies (24,25). This study’s context shows the fusion between African traditional practices and Christianity. While these altruistic motivations stem from cultural and Christian influences, caregivers tend to associate their manifestation more with Christianity. This intricate blend of traditional African practices with Christianity reflects Magesa (56) argument that such practices underlie African religiosity in both Christianity and Islam, thereby shaping the fundamental worldview of most African Christians. These findings suggest that culture and religion play a major role in why study participants assume and continue caregiving.

Again, having a sense of moral responsibility also motivated caregiving. This obligation was attributed to one’s birth order (eg, being the eldest/youngest son/daughter and depending on the primary practice in specific communities), marital relationship with the care recipient, maintaining a family’s caregiving legacy, and unexpected familial circumstances. Previous research has highlighted a sense of caregiving responsibility due to filial obligation and spousal relationship with care recipients (15,17,18). Similarly, family birth order as a reason for caregiving responsibility has been reported among Japanese and Chinese carers, where the eldest son or their wives are designated as primary carers, and among Turkish and Moroccan informal carers, where the eldest daughters or eldest sons’ wives assumed this role (22,28,57,58).

However, this study’s findings differ from previous studies conducted in Chinese, Japanese, Moroccan, and Turkish cultures because birth order was not restricted to eldest daughters or eldest males and their wives. In specific communities in this study, being the youngest son or daughter also conferred caregiving responsibility. Additionally, this finding is distinct from studies conducted in Western countries where the literature suggests the absence of cultural implications regarding birth order and caregiving is motivated by a sense of personal responsibility or other individual factors that shape the caregiving experience (59,60). Furthermore, maintaining one’s family legacy of caregiving adds another layer of distinctness to this finding from the Western gerontology literature, in that informal caregiving is revered in traditional African communities. Therefore, abandoning an immediate or extended family member attracts shame and disregard from the community (30,61).

Also, various unexpected familial situations, including having greater financial resources compared to siblings, proximity to care recipients, and the inadequate performance of caregiving tasks by siblings triggered a sense of responsibility which motivated caregiving. Despite their willingness to assume the caregiving role, these unexpected situations highlighted a degree of unpreparedness and initial hesitation in accepting this responsibility. The situation of unpreparedness/reluctance suggests that these caregivers may potentially experience higher caregiving burdens because the motivations for caregiving could significantly influence the outcome of caregiving for caregivers (22,62).

Additionally, the eagerness for peaceful longevity, a unique finding in this study, emerged as another motivation for informal caregiving. The carers expressed their wish to live long and received assurance of future support from their adult children. This motivation was influenced by cultural and religious beliefs that old age is God’s blessing to good people and a reward for caregiving. This finding stands out because previous studies on religion-informed caregiving motives have mostly focused on Islamic contexts (24,25,28), whereas this study was conducted in the context of Christianity and African traditional practices. The reliance on God for longevity through religion and cultural beliefs reflects Nigeria’s socio-economic factors and lower life expectancy. As the participants anticipate receiving comparable care from their adult children soon, such expectation may hinge on their offspring’s religious and cultural solidarity. Therefore, future studies should explore how religion/culture influences Nigerian youths’ decision to provide informal care to identify any differences compared with older carers.

### Implications

Understanding the motivations for informal caregiving among older adults (50 years and older) has theoretical and practical significance in the Nigerian context. Theoretically, it can contribute to developing new theories/models or enhance our understanding of existing theories about caregiving behavior and the factors influencing such motivations. Practically, this study has implications for developing policies and programs promoting/sustaining the motives for informal caregiving. The first implication is the need for policies strengthening personal and community commitment to informal caregiving. This can be achieved through economic support like tax allowances for carers in civil service, material/financial (stipend) assistance for unemployed or underemployed caregivers, and health care subsidies for all caregivers. Such support provision can motivate individuals’ assumption and continuity in the caring role, sustaining the culture of informal caregiving and facilitating older adults’ expectation for necessary supportive resources from their younger generations.

Also, the familial circumstances that trigger a sense of caregiving responsibility underscore the need for caregiver support groups to provide valuable resources and skills for managing challenging caregiving and family situations. Such intervention should also encourage moral incentives that reflect and preserve respect for informal caregiving and include therapeutic services that address potential psychological impacts resulting from strained family relationships due to caregiving duties. Last, policies for the government to step in when family and community members fail to acquire the caregiving role and function capacity are essential.

## Limitations

The first limitation of our study concerns the homogeneity of the sample, which largely consisted of educated individuals. This was due to specific population characteristics and the sampling strategy adopted in our study location. Initially faced with challenges accessing participants, we resorted to snowball sampling, whereby earlier recruited participants referred other caregivers for inclusion in the study. As Imo state has a high literacy rate (45,46), the initially recruited participants and further referrals were literate individuals. Including a more diverse educational background might have yielded more varied findings. This limitation highlights the need for future studies to ensure proper representation of older caregivers with little or no formal education. Additionally, it should be noted that our study was conducted in a predominantly urban area, and participants primarily identified as Christians. Therefore, caution should be exercised when interpreting the findings as they may not fully apply to all locations within Southeast Nigeria nor to caregivers of Igbo ethnicity who identify as Muslims.

## Conclusion

Our findings identified several motivations for caregiving among older adults aged 54 years and older assisting other older family members and friends in Southeast Nigeria. These motivations included reciprocity, altruism, eagerness for longevity, and a sense of moral responsibility. Although the African collectivist philosophy celebrates altruism, reciprocity seems more dominant in Southeast Nigeria, serving as a preferred means of prioritizing family members’ needs. Our findings have implications for the wider Nigerian society to sustain the culture of informal caregiving. What could be done to foster this collectivist virtue and the necessary remedies to address those who fail/refuse to embrace these values remain a challenge for practitioners, researchers, and policymakers.

## Supplementary Material

igad140_suppl_Supplementary_Material
