# Supplementary material for: Aging and Caring: Exploring Older Adults’ Motivation for Informal Caregiving to Other Aging Individuals in Nigeria
Source: Innov Aging. 2024 Jan 2;8(4):igad140. doi: 10.1093/geroni/igad140 (PMC11020218; doi:10.1093/geroni/igad140)
Supplement: igad140_suppl_Supplementary_Material [file igad140_suppl_supplementary_material.docx]

# **Online Supplementary Material**

**Sample interview guide**

**Introduction and socio-demographics:**

- Can you please tell me your name?
- How old are you?
- What is your marital status?
- What economic activity are you involved in?
- How much do you earn monthly?
- What is your highest education level?
- How would you describe your health status?
- Do you co-reside with your care recipient?
- What is your religious affiliation?
- How are you related to your care recipient?
- How old is your care recipient?
- What is their health status?
- How old are they?

**Questions**

1. Do you remember when you first assumed the role of an informal caregiver to your relation? Please tell me the story of how it started. (Identify the existence of reciprocity, altruism, and other motivational factors).
2. Since then, until now, what constitutes your caregiving responsibility?
3. Let us talk about your caregiving role. Tell me how you see it. What are your thoughts about being an older adult providing care to another ageing individual?
4. Given the state of your relationship with your care recipient, how would you describe the quality of care you are providing? (Probe for reciprocity, altruism, etc.).
